# Optimal Size Criterion for Malignant Lymph Nodes and a Novel Lymph Node Clinical Staging System for Unresectable Esophageal Squamous Cell Carcinoma: Evaluation by Multislice Spiral Computed Tomography

**DOI:** 10.7150/jca.61994

**Published:** 2021-09-03

**Authors:** Hang Wang, Zheng Lin, Yimin Lin, Ruigang Huang, Moliang Qiu, Xiane Peng, Fei He, Liping Huang, Zhisheng Xiang, Wanting Lu, Siyou Yan, Shuang Liu, Huimin Yang, Zhihui Zhang, Zhijian Hu

**Affiliations:** 1 Department of Epidemiology and Health Statistics, Fujian Provincial Key Laboratory of Environment factors and Cancer, School of Public Health, Fujian Medical University, Fuzhou, 350108, China.; 2 Department of Disease Prevention and Healthcare, Fujian Provincial Hospital South Branch & Fujian Provincial Jinshan Hospital, Fuzhou, 350001, China.; 3 Key Laboratory of Ministry of Education for Gastrointestinal Cancer, Fujian Medical University, Fuzhou, 350108, China.; 4 Fujian Digital Institute of Tumor Big Data, Fujian Medical University, Fuzhou, 350122, China.; 5 Fujian Center for ADR monitoring, Fujian Food and Drug Administration, Fuzhou, 350003, China.; 6 Department of Imaging, Zhangzhou Affiliated Hospital of Fujian Medical University, Zhangzhou, 363000, China.; 7 Department of Imaging, Affiliated Fuzhou First Hospital of Fujian Medical University, Fuzhou, 350009, China.

**Keywords:** lymph node metastasis, nonsurgical, prognosis, esophageal squamous cell carcinoma, multislice spiral computed tomography

## Abstract

**Objectives:** The current Chinese draft nodal clinical staging system for unresectable esophageal cancer is controversial. Our study aimed to propose a new diagnostic criterion for lymph node metastasis (LNM) detected by multislice spiral computed tomography (MSCT) in nonsurgically treated esophageal squamous cell carcinoma (ESCC) patients and then develop a novel lymph node (LN) clinical staging system for better individual prognostic prediction.

**Methods:** The short-axis diameters of regional LNs were measured in 393 nonsurgical patients. Regional nodes were considered positive for malignancy if the nodal size exceeded the optimal size, which was determined by Kaplan-Meier survival analysis. The novel LN clinical staging system was then constructed using the LASSO model based on the relative prognostic importance of different LN stations. Validation cohort was included to confirm the prognostic performance.

**Results:** Regional nodes were considered positive for malignancy if they were larger than 10 mm in the low cervical and upper thoracic segments, 7 mm in the middle thoracic segment, and 8 mm in the lower thoracic and celiac segments. Using the LASSO model, stations 2R, 3A, 7 and 16 were qualified in the model. Further analysis showed that our LN clinical staging system had better homogeneity, discriminatory ability and clinical value than the draft nodal staging system.

**Conclusions:** Our results show that the new diagnostic criterion may improve the diagnostic value of MSCT in metastatic LNs. The novel LN clinical staging system can stratify nonsurgically treated ESCC patients into different risk groups, providing valuable information for decision making and outcome prediction.

## Introduction

The accurate diagnosis of lymph node metastasis (LNM) in patients with esophageal squamous cell carcinoma (ESCC) is essential for accurate preoperative staging, prediction of survival, and therapeutic strategy selection. Most studies of LNM diagnosis were pathological, such as the TNM staging system published by the American Joint Committee on Cancer (AJCC) 1. Since the AJCC TNM staging system is based on the results from patients treated with surgery alone, it is still unknown whether it is suitable for patients with locally advanced disease who are clinically unresectable 2.

Computed tomography (CT) scan is the most commonly used noninvasive method to evaluate the metastatic infiltration of lymph nodes (LNs) in ESCC. Unfortunately, there is controversy regarding the definition of an ideal value for LN involvement. Glazer et al. 3 proposed for the first time in 1984 that the short-axis diameter of LNs on CT is much more sensitive than the long-axis diameter and can avoid spatial errors. The presence of enlarged LNs exceeding 10 mm in the short-axis diameter has often been chosen as the standard of CT to diagnose LNM 4, 5. However, normal and metastatic LNs often overlap in size; sometimes, the diameters of metastatic LNs are less than 10 mm, resulting in a low diagnostic accuracy for LNM (38% to 70%) and an underestimation of the N stage 6. Therefore, various authors believed that it is reasonable to reduce the CT diagnostic criterion of the short-axis diameter of positive LNs in ESCC 7-9. Despite China having a greater disease burden than the rest of the world, few studies have been performed in Chinese routine clinical practice until the Clinical Staging Standard for Esophageal Carcinoma Treated with Non-Surgical Methods (draft staging system) was proposed in 2010 10. The general standard for lymphadenectasis in the draft nodal staging system is short-axis diameter of lymph node ≥10 mm and long-axis diameter of paraesophageal, tracheoesophageal sulcus, pericardial and abdominal lymph nodes ≥5 mm. Though the draft staging system has been widely used to stratify patients who cannot undergo surgical operation, it only investigated the diagnostic criterion for LNM by comparing the nodal sizes measured by imaging examination with those obtained by postoperative pathologic findings, so it may not be suitable for unresectable esophageal carcinoma. More than 90% of the pathological type of esophageal cancer in China is the squamous-cell carcinoma type 11, but the draft staging system does not separately classify esophageal carcinoma according to the histopathological cell type, which has an important impact on prognosis. In addition, bidirectional and skip metastasis have been frequently observed to occur in ESCC 12. LNs located in different anatomical zones may not share equal prognostic significance, and the specific criterion for LN involvement lacks high-level evidence, which casts doubt on the utility of the draft nodal staging system in improving individualized therapeutic strategies 13-16. Therefore, it is plausible to connect relatively distant LN stations according to their relative prognostic value and consider them as an entirety.

This study aims to establish a new criterion for estimating the presence of LNM on contrast-enhanced multislice spiral computed tomography (MSCT) images based on the association with patient prognosis. Then, we used the least absolute shrinkage and selection operator (LASSO) model to categorize the metastatic LN stations into dominant and nondominant groups according to their relative prognostic importance to develop a novel LN clinical staging system in order to better predict the survival of nonsurgical patients with ESCC.

## Materials and methods

### Study groups

A total of 393 nonsurgical patients with ESCC were prospectively enrolled in our study between December 2009 and December 2014 at the Department of Thoracic Surgery, Zhang Zhou Hospital, Fujian Province, China. The tumor location (cervical, upper, middle, lower), primary tumor (d-T1, d-T2, d-T3, d-T4), regional LNs (d-N0, d-N1, d-N2) and distant metastases (d-M0, d-M1) were coded according to the draft manual (Table S1).

All patients enrolled in the study met the following criteria: (a) underwent a baseline MSCT scan; (b) received an esophagoscope biopsy followed by pathological diagnosis; and (c) received nonsurgical palliative treatment instead of neoadjuvant or adjuvant treatment according to a standardized protocol for advanced disease or functional inoperability. Patients were excluded if they (a) had nonsquamous cell carcinoma; (b) underwent radical esophagectomy; or (c) had a history or concurrent diagnosis of another type of cancer.

All of the patients included in the current study were randomly divided into the derivation or validation cohorts at an approximately 1:1 ratio. The novel LN clinical staging system was derived from the derivation cohort and validated in the validation cohort. This study was approved by the Ethics Committee of Fujian Medical University. Written informed consent was obtained from all participants.

### Contrast-enhanced MSCT imaging protocols

Contrast-enhanced MSCT scans were performed using a LightSpeed scanner (GE Healthcare). All patients were in the supine position, and the scan images were obtained from the level of the lower neck to the upper abdomen according to the following scanning protocols: 64×0.625 mm^2^ collimation, 0.984 pitch, 5 mm slice width, 1.25-2.5 mm reconstruction increment, 1.25-2.5 mm slice spacing, 60-100 ml injection of intravenous contrast medium at a rate of 2.0-3.0 ml/s at 12 kV and 50-600 mA.

### Image analysis

Regional and nonregional LNs are readily visualized in paraesophageal and retroperitoneal fat. Enlarged LNs and clusters of multiple LNs are abnormal. The LNs were counted, and the shortest diameter of each node in the three-dimensional lymph node reconstruction was considered the short-axis diameter of the LN (Fig S1). Two board-certified radiologists who were blinded to the clinical characteristics independently calculated the nodal size on the MSCT images. The average of the two radiologists' results was used in the analysis for the nodal size. A third radiologist was consulted when the difference of the results was more than 2 mm between the 2 primary radiologists. The nodal size in lung segments (11R, 11L, 12R, 12L, 13R, 13L, 14R, 14L) was not evaluated in this study because there were few LNs involved in the MSCT examination.

The definition of the tumor location was determined from the position of the upper edge of the lesion in the esophagus, which referenced distance from the incisor teeth 1.

### Follow-up

All patients were regularly followed up every 3 months for the first 2 years, every 6 months until 5 years, and then annually at our outpatient clinic or through a telephonic interview. Overall survival (OS) was calculated using the time from the date of the first diagnosis of ESCC to the date of death or the last follow up. Disease-free survival (DFS) was interpreted as the interval between the date of the first diagnosis of ESCC and the date of either disease relapse or death, whichever came first. The deadline of the follow-up was set at December 2016, and the follow-up rate was 98.98%. The cases lost to follow up were treated as censored data for the survival analysis. A total of 304 patients (77.35%) died during the follow-up period.

### Statistical analysis

All analyses were performed using SPSS software version 19.0 (SPSS, Inc., Chicago, IL) and R software version 3.5.0 (Vienna, Austria). Statistical significance was set at 0.05 for two-tailed tests. Kaplan-Meier survival curves were used to estimate the median overall survival and the 1-, 3-, and 5-year OS rates, and the log-rank test was used to assess the survival differences between groups. Multivariate survival analysis was performed using a backward stepwise Cox proportional hazards regression model.

The LN stations were grouped according to the International Association for the Study of Lung Cancer (IASLC) lymph node map (Table S2 and Fig S2). In the current study, we identified the new criterion for LNM in nonsurgical patients with ESCC based on the nodal size, which was sorted by the short-axis diameter (1-mm increments, range 3-10 mm) detected by pretherapy MSCT scans. To determine the optimal size criterion for the detection of LNM, log-rank χ^2^ values from 393 patients in the derivation and validation cohorts were calculated to compare survival differences by stratifying patients based on the enlarged nodal size sorted by the short-axis diameter in each station of the low cervical segment (LCS), upper thoracic segment (UTS), middle thoracic segment (MTS), lower thoracic segment (LTS) and celiac segment (CS). Next, we used the LASSO Cox regression model to select the most useful prognostic LN stations out of all the unresectable ESCC-associated LN stations and then constructed the novel LN clinical staging for individual prognostic prediction.

The likelihood ratio χ^2^ test related to the Cox regression model was used to measure the homogeneity. To compare the discriminatory ability of different staging systems, we assessed both the Akaike information criterion (AIC) value and the time-dependent receiver operating characteristic (ROC) curve. A smaller AIC value indicates a better model for predicting outcome. Decision curve analysis was used to evaluate the practical clinical value of the staging system by quantifying the net benefits 17. The average deviation about the probability threshold (ADAPT), which is a more recently developed index to measure the utility of a prediction model, was also performed in our study 18.

## Results

### Characteristics of the Study Groups

After randomization, the derivation and validation cohorts contained 197 and 196 patients, respectively. The detailed clinicopathological characteristics of the patients are listed in Table 1. In brief, the majority of patients were male (n=304, 77.35%), and the middle thoracic esophagus (MTE) was most often involved (n=228, 58.02%). The location of the ESCC lesion was the cervical esophagus in 18 patients, the upper thoracic esophagus in 115 patients, the middle thoracic esophagus in 228 patients, and the lower thoracic esophagus in 32 patients.

Among the 393 patients evaluable, the median follow-up duration for OS was 483 days, and the 1-, 3- and 5-year OS rates were 61.28%, 35.12% and 16.15%, respectively. A total of 12,866 LNs were recorded from 393 patients according to the draft regional LN definition on MSCT images and were submitted for metastatic LN detection (Fig. S3). The incidence of LNM according to the draft diagnosis criterion for LNM was 98.73% (388 of 393 cases). The prevalence of positive lymph nodes (PLNs) in varying nodal stations separated by tumor location is presented in Fig. S4.

### Criterion for the diagnosis of LNM on MSCT

**Figure 1:**
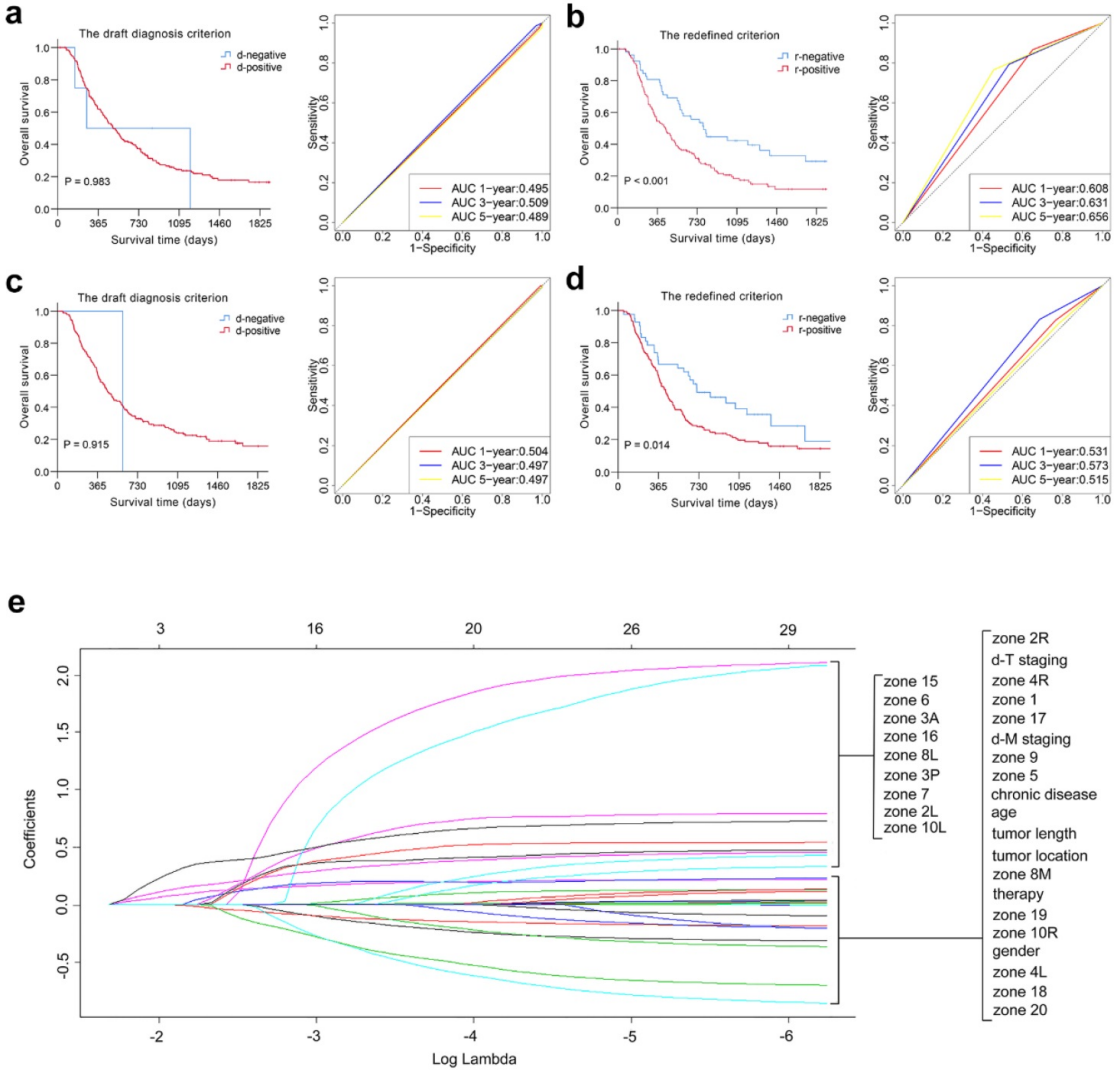
Overall predictive performance comparison of two diagnosis criteria for LNM was measured by the survival analysis and the time-dependent ROC curves in derivation (a, b) and validation cohorts (c, d). Plots (e) show the LASSO coefficient profiles of the 21 ESCC prognosis-associated LN stations based on the redefined criterion. A vertical line is drawn at the value (log = -2.239) chosen by 5-fold cross-validation.

The distribution of the examined LNs for 393 patients and log-rank χ^2^ values at different cut-off points of the short-axis diameter in each station and segment are listed in Table S3 and Table 2. The prognostic value of various nodal sizes in each segment was quantified using the sum of the log-rank χ^2^ values of each station, and a higher value indicates perfect discrimination. Thus, regional nodes were considered positive for malignancy in our study if they were larger than 10 mm in the LCS and UTS, 7 mm in the MTS, and 8 mm in the LTS and CS (redefined criterion). The detailed differences between the draft diagnosis criterion and the redefined criterion are described in Table 3. The new criterion for the LNM of 89 patients was changed from the draft diagnosis criterion for LNM, as shown in Table S4. Kaplan-Meier plots showed that the category-redefined criterion had better discriminatory ability than the draft diagnosis criterion for LNM in both the derivation and validation cohorts (*P* < 0.05). Furthermore, ROC analysis presented satisfactory results for our redefined criterion compared with the draft diagnosis criterion for LNM, as shown in Fig. 1 and Fig. S5.

### Development of the novel LN clinical staging system using the LASSO Cox regression model

Based on the redefined criterion for LNM, we developed a novel LN clinical staging system in this study. According to the multivariate LASSO Cox regression result in the derivation cohort, stations 2R, 3A, 7 and 16 were qualified in the model, and they were grouped as the dominant lymph node stations (DLNS), while the other 17 regional LN stations were grouped as the nondominant lymph node stations (N-DLNS) (Fig. 1 and Fig. S6). All patients were categorized into the following three groups to develop the novel LN clinical staging system according to the LNM status: node-negative patients (n-N0, n=94), metastasis in N-DLNS (n-N1, n=112), and metastasis in DLNS or both positive (n-N2, n=187). The comparisons of the draft nodal staging system and the novel LN clinical staging system are listed in Table 3. The proportion of patients who migrated between stages when the novel LN clinical staging system was applied is summarized in Table S5.

**Figure 2:**
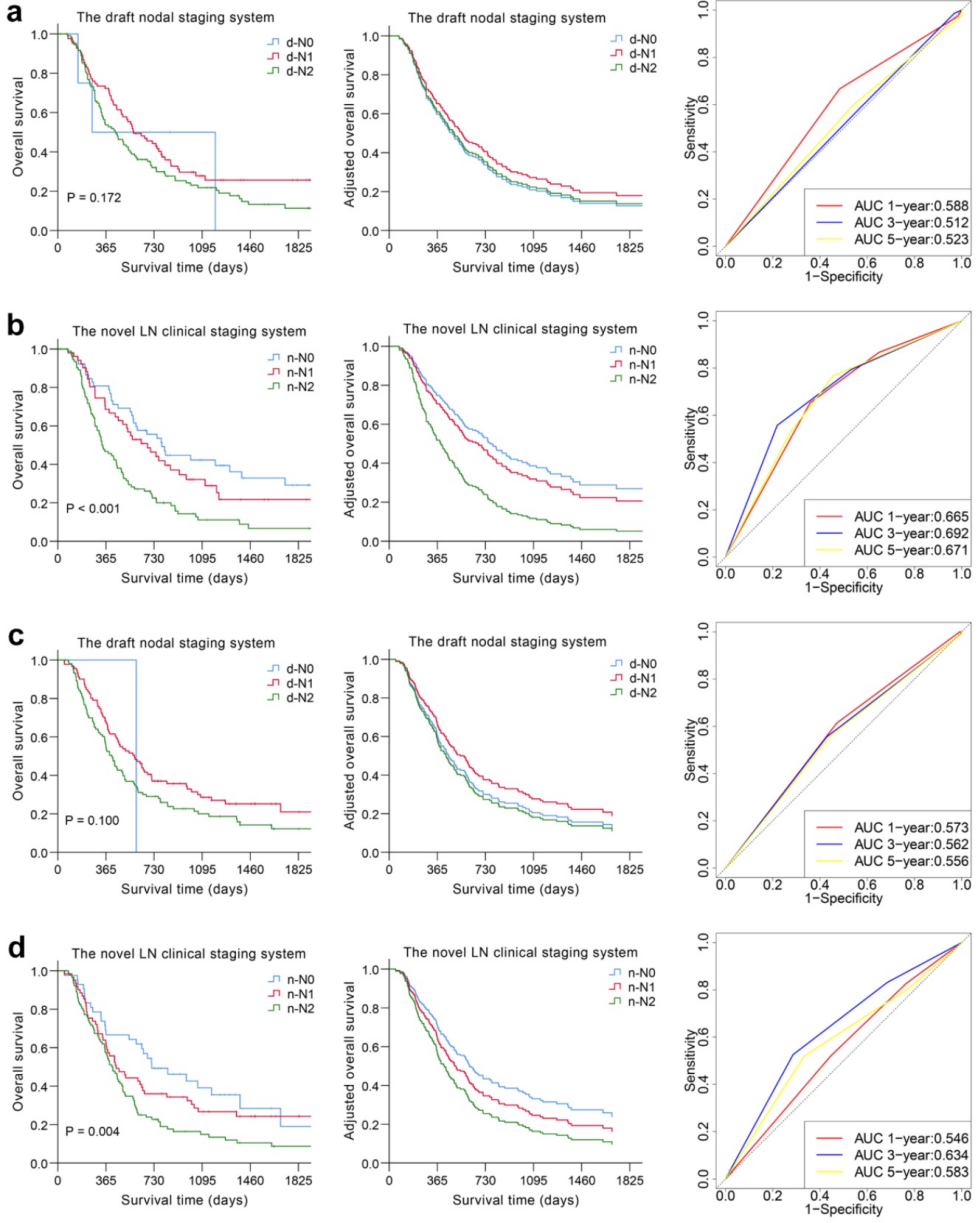
Overall predictive performance comparison of two clinical staging systems was measured by the survival analysis and the time-dependent ROC curves in derivation (a, b) and validation cohorts (c, d).

Kaplan-Meier survival distributions in the derivation cohort suggested that the novel LN clinical staging system has good discriminatory ability. Compared with the draft nodal staging system, the novel LN clinical staging system showed an improvement in the separation of each substage (*P* < 0.05) (Fig. 2 and Fig. S5). Then, 2 separate multivariate models were constructed using Cox regression analysis after adjusting for clinicopathological variables in the derivation cohort, one with the draft nodal staging system and the other with the novel LN clinical staging system. In multivariate analysis, the novel LN clinical staging system was shown to be an independent predictor of OS, while the draft nodal staging system was not (Table 4 and Fig. 2). We assessed the prognostic accuracy of the novel LN clinical staging system using time-dependent ROC analysis at different follow-up times. ROC analysis showed higher sensitivity and specificity with the novel LN clinical staging system for predicting OS and DFS than that with the draft nodal staging system at 1, 3, and 5 years, as shown in Fig. 2 and Fig. S5.

### Validation and comparison of the prognostic performance between the novel and draft LN clinical staging systems

We used the novel LN clinical staging system to predict tumor prognosis in the validation cohort. The Kaplan-Meier curves are depicted in Fig. 2 and Fig. S5. The overall and disease-free survival curves could easily be distinguished according to the novel LN clinical staging system (*P* = 0.004, *P* = 0.007), whereas for the N category based on the draft nodal staging system, the result was not satisfactory (*P* = 0.100, *P* = 0.218). The Cox model and ROC analysis also revealed better survival predictive ability for the novel LN clinical staging system than for the draft nodal staging system (Table 4, Fig. 2 and Fig. S5).

**Figure 3:**
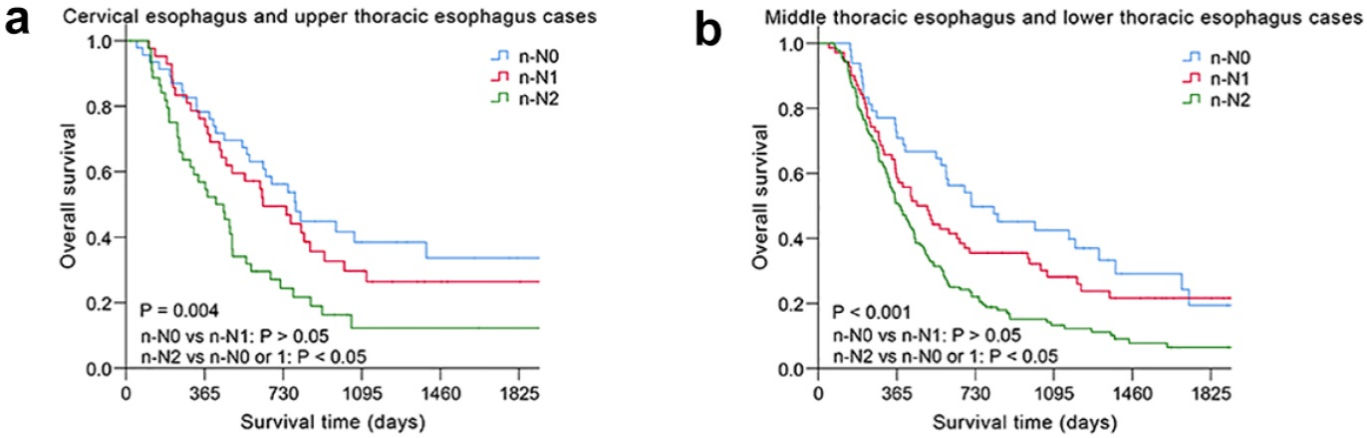
Kaplan-Meier survival analysis of overall survival according to the novel LN clinical staging system in subgroups of nonsurgical patients with ESCC in different tumor location. (a) Cervical esophagus and upper thoracic esophagus cases (n=133). (b) Middle thoracic esophagus and lower thoracic esophagus cases (n=260).

**Figure 4:**
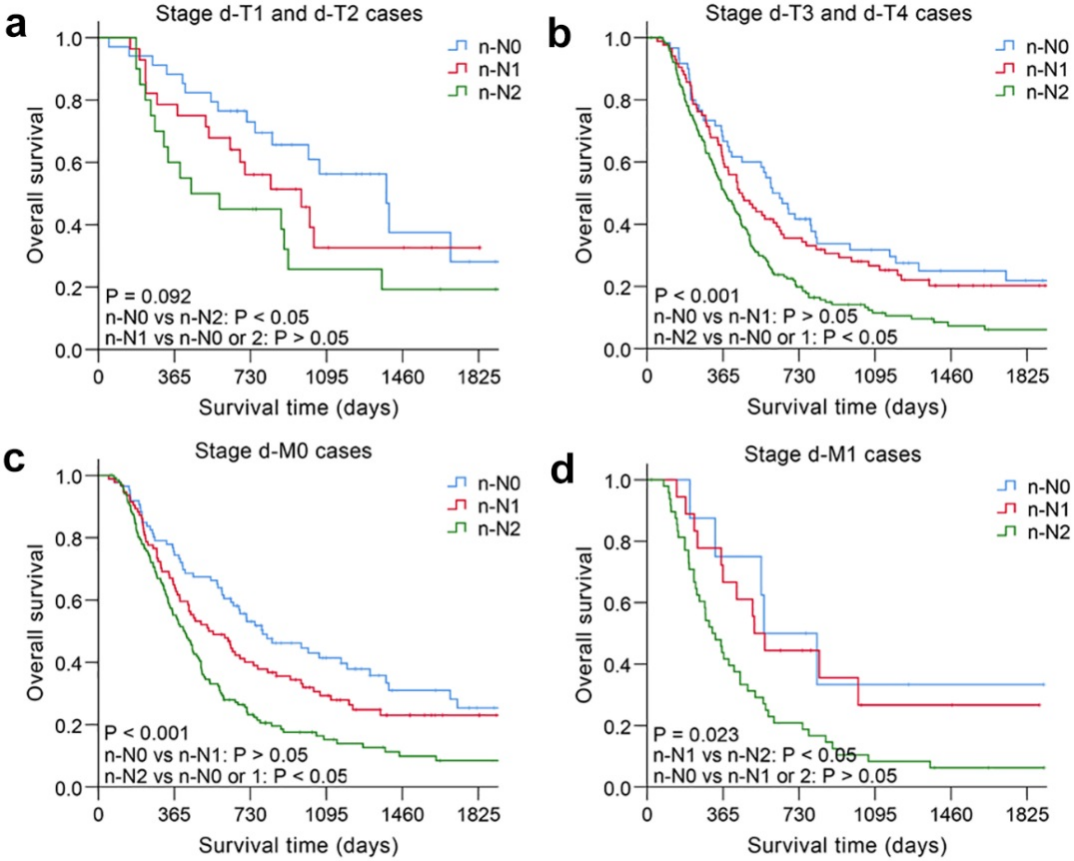
Kaplan-Meier survival analysis of overall survival according to the novel LN clinical staging system in subgroups of nonsurgical patients with ESCC in different cancer stages. (a) Draft primary tumor stage 1/2 (n=82). (b) Draft primary tumor stage 3/4 (n=311). (c) Draft distant metastasis stage 0 (n=318). (d) Draft distant metastasis stage 1 (n=75).

The nodal category was reclassified in nonsurgical patients with ESCC by combining the most useful prognostic LN stations, and the novel LN clinical staging system showed high performance with good discrimination and homogeneity in the validation cohort (Table 5). The performance of this novel LN clinical staging system was also assessed in ESCC subgroups. When stratified by tumor location, there were significant survival differences among the three groups (n-N0, n-N1, n-N2) for cervical esophagus (CE)/upper thoracic esophagus (UTE) cases and middle thoracic esophagus (MTE)/lower thoracic esophagus (LTE) cases, respectively, *P* < 0.05 (Fig. 3). Moreover, the novel LN clinical staging system could also classify survival for patients with different cancer stages (draft primary tumor stage 3/4 and draft distant metastasis stage 0/1, Fig. 4, respectively, *P* < 0.05). Finally, the clinical usefulness of the novel LN clinical staging system was evaluated using decision curve analysis and ADAPT curves by quantifying the net benefits (Fig. S7 and Fig. S8). The new staging system seems to have better net benefits compared with the draft nodal staging system across all threshold probabilities. Overfitting was corrected by carrying out a total of N=500 bootstrap replicates, and the result was acceptable. Confidence intervals and *P* values for the comparison of the two clinical staging systems were also calculated.

## Discussion

Those who is elder, in advanced stage or cannot tolerate surgery may be used the nonsurgical treatment method to control the tumor progression. In this study, we developed a novel LN clinical staging system to predict the prognosis of nonsurgical patients with ESCC. The revision for our staging system compared to the draft nodal staging system consisted of changes in the N descriptors that reclassified regional LN involvement by evaluating different prognostic values of various nodal sizes in each station and segment measured by MSCT and proposed a novel approach to merge some LN stations according to their relative importance regarding their association with survival and categorize them into DLNS (stations 2R, 3A, 7 and 16) and N-DLNS (the other LN stations). Metastases involved in DLNS were verified for their prognostic value in predicting survival. Further statistical analysis demonstrated that the novel LN clinical staging system was an independent prognostic predictor for long-term survival with good performance of practical clinical value, discrimination ability and homogeneity, which could provide better stratification for nonsurgically treated Chinese ESCC patients with different prognoses compared with the draft nodal staging system.

ESCC is an aggressive disease associated with a high frequency of LNM compared to other gastrointestinal malignancies 19, 20. LNs located in different anatomical zones may not share equal prognostic significance because the presence of stepwise and skipped metastasis are both common in ESCC, which allowed us to connect relatively distant LN stations 21. We previously published a novel approach to categorize regional LN stations into dominant and nondominant groups according to their relative prognostic importance and to examine the feasibility and utility of this classification method in predicting the prognosis of surgical ESCC patients 22. However, the majority of ESCC patients have locally advanced disease when they are diagnosed, and more than half of the patients with locally advanced disease are clinically unresectable; thus, it is still an issue worthy of further reflection and inquiry.

Nodal involvement in ESCC is often diagnosed by the size of the nodes, and the optimal size criterion for the detection of malignant LNs remains controversial. Spiral CT scans are the most important noninvasive diagnostic method for ESCC and LN staging and can clearly show the size, number and anatomical location of the enlarged LNs and determine the staging of LNs combined with morphological changes. In contrast with previous studies, the LNs displayed in MSCT were evaluated as having relative prognostic value for nonsurgically treated ESCC patients based on the short-axis diameter in our research. We assessed the relative prognostic importance of nodal size in each station and segment measured by MSCT, making comparisons with the draft diagnosis criterion for LNM. Our results indicated that the best diagnostic cut-off points for the regional nodes were 10 mm in LCS and UTS, 7 mm in MTS, and 8 mm in LTS and CS. In contrast to the survival curves of the draft diagnosis criterion, we found that curves stratified according to our redefined criterion did not overlap.

Accurate prognostic assessment is essential for the selection of appropriate therapy. China was the first to propose draft staging system for esophageal carcinoma treated with nonsurgical methods, and similar drafts currently exist in many other countries. Although the current draft staging system provides a great improvement over previous editions with the N category by stratifying patients based on the location of the metastatic lesions involved, several studies have shown that this staging system has several limitations, mainly related to the large heterogeneity of N1 and N2 patients 23-25. In this study, we also found that the survival differences between the d-N1 and d-N2 categories were not significant in predicting long-term survival. The definition of regional LNs in the draft nodal staging system did not involve supraclavicular nodes for upper thoracic esophagus cases and left gastric nodes for upper/middle thoracic esophagus cases, their prognostic value need to be further evaluated. In addition, the draft staging system does not cover the histopathological cell type, which is significantly related to the prognosis of the disease. And previous studies have reported that tumor location can influence the extent of LNM 26, 27. Squamous cell carcinoma is more frequent in the proximal to middle esophagus, whereas majority of adenocarcinoma lesions are found in the distal esophagus 28. Consequently, the stage needs to be classified separately according to the histopathological cell type, for example, ESCC, which is the most common pathological type of esophageal cancer in China. In our study, we used the LASSO Cox regression model to categorize regional LN stations into dominant and nondominant groups according to their relative prognostic importance to develop a novel LN clinical staging system for unresectable ESCC. With the comparisons of the draft staging system, there were obvious survival differences in each substage of our staging system.

There are several potential limitations of our study. First, although all of these data were assembled from the hospital most well known in the area for the treatment of ESCC, this is a single institutional study, which may make the results from our study not generalizable to other populations. Thus, a multicenter collaborative prospective study is needed to substantiate our results. Second, the diagnosis of LNM based solely on minor axis diameters is not sufficient to provide a reliable pretreatment evaluation of ESCC. Similar results have been reported for lung cancer, colon cancer and pancreatic cancer. More diagnostic indicators therefore need to be identified to improve the diagnostic value of MSCT for metastatic LNs of unresectable ESCC. Third, the distribution of tumor location may also impact the extent of LNM. We studied relatively few cervical esophagus (CE) and lower thoracic esophagus (LTE) cases, which could affect our conclusions. In a recently published large-scale study, a good relationship was seen between the tumor location and LNM regions 29. However, the application of the LASSO Cox regression analysis in this study could partly reduce the instability during modeling. Finally, as this study is focused on unresectable ESCC, our proposed staging system is not compared with the pathological staging. Since histopathology is a gold standard for the diagnosis of LNM, the results of pathological diagnosis need to be used as a comparison standard or reference for our staging system in the future in order to determine its significance in clinical practice.

In conclusion, we proposed a new diagnostic criterion for the LNM of unresectable ESCC based on the short-axis nodal size measured by MSCT. Then, we reclassified the nodal category by assessing the prognostic value differences of regional LN stations with a straightforward and clinically applicable procedure. To facilitate clinical utilization, further validation in multicenter or large-scale studies is warranted.

## Supplementary Material

Supplementary figures and tables.

## Figures and Tables

**Table 1:** Baseline characteristics of patients in the derivation and validation cohorts

Variable	Derivation cohort (n = 197)	Validation cohort (n = 196)	*P* value
**Gender**			0.697
Male	154 (78.2%)	150 (76.5%)	
Female	43 (21.8%)	46 (23.5%)	
**Age (years)**			0.805
≤60	82 (41.6%)	84 (42.9%)	
>60	115 (58.4%)	112 (57.1%)	
**Chronic disease**			0.029
Yes	76 (38.6%)	97 (49.5%)	
No	121 (61.4%)	99 (50.5%)	
**Tumor location**			0.772
CE	11 (5.6%)	7 (3.6%)	
UTE	55 (27.9%)	60 (30.6%)	
MTE	115 (58.4%)	113 (57.7%)	
LTE	16 (8.1%)	16 (8.2%)	
**Histologic grade^a^**			0.825
G1	32 (16.2%)	27 (13.8%)	
G2	70 (35.5%)	75 (38.3%)	
G3	36 (18.3%)	32 (16.3%)	
Missing	59 (29.9%)	62 (31.6%)	
**Draft primary tumor (d-T)**			0.784
d-T1 + d-T2	40 (20.3%)	42 (21.4%)	
d-T3 + d-T4	157 (79.7%)	154 (78.6%)	
**Draft regional lymph nodes (d-N)**			0.292
d-N0	4 (2.0%)	1 (0.5%)	
d-N1	83 (42.1%)	91 (46.4%)	
d-N2	110 (55.8%)	104 (53.1%)	
**Draft distant metastasis (d-M)**			0.165
d-M0	154 (78.2%)	164 (83.7%)	
d-M1	43 (21.8%)	32 (16.3%)	
**Draft staging system**			0.361
I + II	23 (11.7%)	29 (14.8%)	
III + IV	174 (88.3%)	167 (85.2%)	
**Therapy**			0.974
Chemotherapy/Radiotherapy	140 (71.1%)	139 (70.9%)	
Chemoradiotherapy	57 (28.9%)	57 (29.1%)	
**X-ray Tumor length (cm)**			0.791
Median (P_25_,P_75_)	5.50 (4.15~6.67)	5.46 (4.10~6.78)	

CE, cervical esophagus; UTE, upper thoracic esophagus; MTE, middle thoracic esophagus; LTE, lower thoracic esophagus. ^a^ One hundred and twenty-one (30.8%) patients did not have Histologicgrade information.

**Table 2:** Log-rank χ^2^ values at different cut-off points of the short-axis diameter in each station and segment

Nodal size (mm)	LCS + UTS										MTS					LTS				CS					
	1	2R	2L	3A	3P	4R	4L	5	6	Total	7	8M	10R	10L	Total	8L	9	15	Total	16	17	18	19	20	Total
3	0.298	1.129	0.087	0.331	3.247	0.001	0.014	0.150	5.129	10.386	0.698	1.379	2.475	3.039	7.591	0.055	1.593	3.808	5.456	5.620	0	0.114	0.731	0.001	6.466
4	2.699	0.131	0.016	0.661	3.707	0.001	0.981	0.461	1.673	10.330	2.220	1.088	3.498	4.502	11.308	0.659	1.428	0.153	2.240	8.363	0.004	0.058	0.424	0	8.849
5	2.036	0.606	0.829	2.156	2.911	0.133	0.287	0.127	0.019	9.104	0.875	1.785	1.111	2.636	6.407	0.459	2.717	0.002	3.178	8.815	0.917	0.404	0.087	0.092	10.315
6	2.877	4.213	0.407	2.603	2.759	0.873	0.108	0.211	0.022	14.073	3.292	2.878	0.617	4.143	10.930	1.427	0.566	0.01	2.003	7.867	3.068	1.295	0.270	0.589	13.089
7	2.020	4.743	1.717	1.472	8.474	0.130	0.031	0.414	0.867	19.868	9.816	1.595	0.009	0.680	12.100	1.794	0.272	0.078	2.144	4.745	1.919	0.823	0.264	0.014	7.765
8	4.084	7.331	2.174	0.480	13.659	3.122	0.275	2.326	0.911	34.362	3.472	0.358	1.572	0.039	5.441	5.698	0.013	7.776	13.487	13.994	1.956	1.520	0.264	0.061	17.795
9	3.602	4.332	0.897	0.480	10.523	4.042	0.080	0.247	30.349	54.552	4.468	0.224	3.788	1.441	9.921	4.273	0.225	5.825	10.323	8.895	1.689	1.044	0	0.183	11.811
10	3.328	5.978	1.349	9.457	6.242	2.686	0.030	2.066	30.349	61.485	2.981	0.625	2.679	-	6.285	4.273	1.099	-	5.372	11.261	1.223	1.864	0	0.057	14.405

LCS, low cervical segment; UTS, upper thoracic segment; MTS, middle thoracic segment; LTS, lower thoracic segment; CS, celiac segment.

**Table 3:** Comparison between the draft nodal staging system and the novel LN clinical staging system

Variable	the draft nodal staging system	the novel LN clinical staging system
Diagnosis criterion for LNM	The general standard is the short-axis diameter of lymph node ≥10 mm; the long-axis diameter of paraesophageal lymph node, and lymph node in tracheoesophageal sulcus and pericardial lymph node ≥5 mm, and abdominal lymph node ≥5 mm	The short-axis diameter of lymph node ≥10 mm in the low cervical and upper thoracic segments, ≥7 mm in the middle thoracic segment, and ≥8 mm in the lower thoracic and celiac segments.
**N Stage**		
N0	No enlargement of lymph node	No enlargement of lymph node
N1	Enlargement of lymph nodes in chest (paraesophageal and mediastinum), carcinoma of inferior segment of oesophagus with left gastric lymphadenectasis, carcinoma of cervical portion of oesophagus with enlargement of supraclavicular lymph nodes	Enlargement of lymph nodes in N-DLNS
N2	Carcinoma of the middle and lower thoracic oesophagus with enlargement of supraclavicular lymph nodes, carcinoma of any segment of oesophagus with enlargement of abdominal para-aortic lymph nodes	Enlargement of lymph nodes in DLNS or both positive in N-DLNS and DLNS

LNM, lymph node metastasis; N-DLNS, nondominant lymph node stations (stations 1, 2L,3P, 4R, 4L, 5, 6, 8M, 8L, 9, 10R, 10L, 15, 17, 18, 19 and 20); DLNS, dominant lymph node stations (stations 2R, 3A, 7 and 16).

**Table 4:** Multivariate survival analysis of the draft nodal staging system and the novel LN clinical staging system

Variable	HR (95% CI)	*P* value
**Multivariate model with the draft nodal staging system^a^**		
***Derivation cohort (n = 197)***		
Gender	0.711 (0.464~1.090)	0.118
Age	1.239 (0.869~1.767)	0.236
**Tumor location**		
CE	1	
UTE	1.125 (0.471~2.687)	0.791
MTE	1.518 (0.641~3.596)	0.342
LTE	1.034 (0.366~2.916)	0.950
**Draft primary tumor (d-T)**		
d-T1 + d-T2	1	
d-T3 + d-T4	1.968 (1.171~3.307)	0.011
Therapy		
Chemotherapy/Radiotherapy	1	
Chemoradiotherapy	0.808 (0.547~1.193)	0.284
X-ray Tumor length (cm)	0.970 (0.871~1.081)	0.586
**the draft nodal staging system**		
d-N0	1	
d-N1	0.832 (0.229~3.028)	0.780
d-N2	0.960 (0.274~3.359)	0.949
**Validation cohort (n = 196)**		
Gender	0.776 (0.510~1.180)	0.236
Age	0.982 (0.697~1.383)	0.915
**Tumor location**		
CE	1	
UTE	0.522 (0.214~1.273)	0.153
MTE	0.561 (0.237~1.329)	0.189
LTE	0.507 (0.180~1.429)	0.199
**Draft primary tumor (d-T)**		
d-T1 + d-T2	1	
d-T3 + d-T4	1.969 (1.173~3.306)	0.010
**Therapy**		
Chemotherapy/Radiotherapy	1	
Chemoradiotherapy	0.751 (0.520~1.085)	0.127
X-ray Tumor length (cm)	1.034 (0.944~1.133)	0.471
**the draft nodal staging system**		
d-N0	1	
d-N1	0.810 (0.107~6.137)	0.839
d-N2	1.075 (0.144~8.005)	0.944
**Multivariate model with the novel LN clinical staging system^b^**		
***Derivation cohort (n = 197)***		
Gender	0.720 (0.473~1.096)	0.126
Age	1.426 (0.997~2.040)	0.052
**Tumor location**		
CE	1	
UTE	1.141 (0.477~2.730)	0.767
MTE	1.423 (0.615~3.291)	0.410
LTE	1.070 (0.394~2.908)	0.894
**Draft primary tumor (d-T)**		
d-T1 + d-T2	1	
d-T3 + d-T4	1.711 (1.013~2.889)	0.044
**Therapy**		
Chemotherapy/Radiotherapy	1	
Chemoradiotherapy	0.766 (0.522~1.123)	0.172
X-ray Tumor length (cm)	0.961 (0.867~1.065)	0.447
**the novel LN clinical staging system**		
n-N0	1	
n-N1	1.203 (0.744~1.946)	0.451
n-N2	2.259 (1.457~3.504)	< 0.001
**Validation cohort (n = 196)**		
Gender	0.852 (0.564~1.288)	0.448
Age	0.933 (0.666~1.308)	0.688
**Tumor location**		
CE	1	
UTE	0.495 (0.201~1.216)	0.125
MTE	0.557 0.233~1.329)	0.187
LTE	0.542 (0.192~1.531)	0.248
**Draft primary tumor (d-T)**		
d-T1 + d-T2	1	
d-T3 + d-T4	1.823 (1.078~3.085)	0.025
**Therapy**		
**Chemotherapy / Radiotherapy**	1	
Chemoradiotherapy	0.739 (0.512~1.066)	0.105
X-ray Tumor length (cm)	1.033 (0.943~1.131)	0.484
**the novel LN clinical staging system**		
n-N0	1	
n-N1	1.270 (0.771~2.092)	0.347
n-N2	1.639 (1.030~2.608)	0.037

CE, cervical esophagus; UTE, upper thoracic esophagus; MTE, middle thoracic esophagus; LTE, lower thoracic esophagus. ^a, b^ Gender (male, female), age (≤60, >60), location of tumor (CE, UTE, MTE, LTE), draft T categories (d-T1 + d-T2, d-T3 + d-T4), therapy (chemotherapy/radiotherapy, chemoradiotherapy), tumor length measured by X-ray (as continuous) were included as covariates in Cox regression model.

**Table 5:** Comparison of the performance of the draft nodal staging system and the novel LN clinical staging system

Model	Figure	Substage	LR χ^2^	AIC Value
**Derivation cohort (n = 197)**				
the draft nodal staging system	2a, S5b	d-N0, d-N1, d-N2	17.64	1389.251
the novel LN clinical staging system	2b, S5d	n-N0, n-N1, n-N2	34.69	1372.202
**Validation cohort (n = 196)**				
the draft nodal staging system	2c, S5f	d-N0, d-N1, d-N2	23.27	1409.259
the novel LN clinical staging system	2d, S5h	n-N0, n-N1, n-N2	25.53	1406.999

AIC, Akaike information criterion; LR, likelihood ratio.

## Abbreviations

LNM: lymph node metastasis
ESCC: esophageal squamous cell carcinoma
CT: computed tomography
LN: lymph node
Draft staging system: Clinical Staging Standard for Esophageal Carcinoma Treated with Non-Surgical Methods
MSCT: multi-slice spiral computer tomography
LASSO: the least absolute shrinkage and selection operator model
d-N: the draft nodal staging
n-N: the novel LN clinical staging
OS: overall survival
DFS: Disease-free survival
HR: hazard ratio
CE: cervical esophagus
UTE: upper thoracic esophagus
MTE: middle thoracic esophagus
LTE: lower thoracic esophagus
LCS: low cervical segment
UTS: upper thoracic segment
MTS: middle thoracic segment
LTS: lower thoracic segment
CS: celiac segment
AIC: Akaike information criterion value
ROC: receiver operating characteristic curve
ADAPT: average deviation about the probability threshold
