# Supplementary material for: Optimal Size Criterion for Malignant Lymph Nodes and a Novel Lymph Node Clinical Staging System for Unresectable Esophageal Squamous Cell Carcinoma: Evaluation by Multislice Spiral Computed Tomography
Source: J Cancer. 2021 Sep 3;12(21):6454–64. doi: 10.7150/jca.61994 (PMC8489143; doi:10.7150/jca.61994)
Supplement: Supplementary file 1 — Supplementary figures and tables. [file jcav12p6454s1.pdf]

Supplementary Tables

Table S1. Clinical Staging Standard for Esophageal Carcinoma Treated with Non-Surgical Methods (draft staging system)

Staging	Description
Draft primary tumor (d-T)	
d-T1	Lesion length in barium meal ≤3 cm and the diameter of oesophageal section with the largest lesion in CT ≤2 cm, and no involvement of adjacent structures
d-T2	Lesion length in barium meal >3-5 cm, and the diameter of oesophageal section with the largest lesion in CT >2-4 cm, and no involvement of adjacent structures
d-T3	Lesion length in barium meal >5-7 cm, and the diameter of oesophageal section with the largest lesion in CT >4 cm, and no involvement of adjacent structures
d-T4	Lesion length in barium meal >7 cm, and the diameter of oesophageal section with the largest lesion in CT >4 cm, with involvement of adjacent structures (including trachea, bronchus, aorta, and pericardium)
Draft regional lymph nodes (d-N)	
d-N0	No enlargement of lymph node
d-N1	Enlargement of lymph nodes in chest (paraesophageal and mediastinum), carcinoma of inferior segment of oesophagus with left gastric lymphadenectasis, carcinoma of cervical portion of oesophagus with enlargement of supraclavicular lymph nodes
d-N2	Carcinoma of the middle and lower thoracic oesophagus with enlargement of supraclavicular lymph nodes, carcinoma of any segment of oesophagus with enlargement of abdominal para-aortic lymph nodes
Draft distant metastasis (d-M)	
d-M0	No distant metastasis
d-M1	Distant metastasis

Lymphadenectasis is the criterion for cancerous metastasis; the general standard is the short-axis diameter of lymph node ≥10 mm, the long-axis diameter of paraesophageal lymph node, and lymph node in tracheoesophageal sulcus and pericardial lymph node ≥5 mm, and abdominal lymph node ≥5 mm

Table S2. LN stations codes and the corresponding regional nodal stations

Station	AJCC regional LN stations	Station	AJCC regional LN stations
1	Supraclavicular nodes	8L	Lower paraesophageal nodes
2R	Right upper paratracheal nodes	9	Pulmonary ligament nodes
2L	Left upper paratracheal nodes	10R	Right tracheobronchial nodes
3A	Pre-vascular nodes	10L	Left tracheobronchial nodes
3P	Posterior mediastinal nodes	15	Diaphragmatic nodes
4R	Right lower paratracheal nodes	16	Paracardial nodes
4L	Left lower paratracheal nodes	17	Left gastric nodes
5	Aortopulmonary nodes	18	Common hepatic nodes
6	Anterior mediastinal nodes	19	Splenic nodes
7	Subcarinal nodes	20	Celiac node
8M	Middle paraesophageal nodes		

Table S3. Distribution of examined lymph nodes for 393 patients in each station and segment

Nodal size (mm)	LCS + UTS									MTS				LTS			CS				
	1	2R	2L	3A	3P	4R	4L	5	6	7	8M	10R	10L	8L	9	15	16	17	18	19	20
< 3	1074 (32.55%)	341 (34.80%)	106 (27.68%)	119 (29.31%)	15 (9.68%)	437 (26.71%)	276 (24.30%)	75 (16.41%)	28 (19.86%)	91 (7.09%)	58 (8.16%)	35 (8.29%)	3 (4.76%)	14 (11.48%)	3 (4.69%)	29 (32.58%)	34 (16.19%)	66 (10.63%)	12 (6.94%)	13 (18.57%)	37 (12.42%)
⩾3	826 (25.03%)	253 (25.82%)	85 (22.19%)	113 (27.83%)	27 (17.42%)	408 (24.94%)	299 (26.32%)	133 (29.10%)	54 (38.30%)	211 (16.45%)	131 (18.42%)	63 (14.93%)	12 (19.05%)	21 (17.21%)	18 (28.13%)	30 (33.71%)	36 (17.14%)	116 (18.68%)	28 (16.18%)	15 (21.43%)	58 (19.46%)
⩾4	492 (14.91%)	185 (18.88%)	76 (19.84%)	78 (19.21%)	39 (25.16%)	314 (19.19%)	242 (21.30%)	113 (24.73%)	28 (19.86%)	285 (22.21%)	151 (21.24%)	94 (22.27%)	11 (17.46%)	29 (23.77%)	13 (20.31%)	16 (17.98%)	33 (15.71%)	121 (19.48%)	38 (21.97%)	14 (20.00%)	69 (23.15%)
⩾5	282 (8.55%)	71 (7.24%)	34 (8.88%)	33 (8.13%)	16 (10.32%)	144 (8.80%)	132 (11.62%)	42 (9.19%)	12 (8.51%)	232 (18.08%)	96 (13.50%)	69 (16.35%)	14 (22.22%)	17 (13.93%)	9 (14.06%)	4 (4.49%)	23 (10.95%)	102 (16.43%)	26 (15.03%)	7 (10.00%)	40 (13.42%)
⩾6	129 (3.91%)	38 (3.88%)	20 (5.22%)	12 (2.96%)	13 (8.39%)	104 (6.36%)	62 (5.46%)	30 (6.56%)	7 (4.96%)	128 (9.98%)	67 (9.42%)	56 (13.27%)	10 (15.87%)	10 (8.20%)	5 7.81%)	3 (3.37%)	17 (8.10%)	46 (7.41%)	23 (13.29%)	10 (14.29%)	24 (8.05%)
⩾7	139 (4.21%)	30 (3.06%)	17 (4.44%)	20 (4.93%)	12 (7.74%)	93 (5.68%)	56 (4.93%)	31 (6.78%)	3 (2.13%)	109 (8.50%)	68 (9.56%)	37 (8.77%)	6 (9.52%)	7 (5.74%)	7 (10.94%)	5 (5.62%)	13 (6.19%)	48 (7.73%)	13 (7.51%)	2 (2.86%)	14 (4.70%)
⩾8	70 (2.12%)	16 (1.63%)	10 (2.61%)	5 (1.23%)	5 (3.23%)	48 (2.93%)	15 (1.32%)	13 (2.84%)	4 (2.84%)	70 (5.46%)	37 (5.20%)	24 (5.69%)	2 (3.17%)	4 (3.28%)	3 (4.69%)	1 (1.12%)	11 (5.24%)	24 (3.86%)	7 (4.05%)	2 (2.86%)	8 (2.68%)
⩾9	61 (1.85%)	2 (0.20%)	7 (1.83%)	7 (1.72%)	7 (4.52%)	31 (1.89%)	15 (1.32%)	8 (1.75%)	1 (0.71%)	51 (3.98%)	27 (3.80%)	9 (2.13%)	3 (4.76%)	2 (1.64%)	3 (4.69%)	1 (1.12%)	10 (4.76%)	24 (3.86%)	6 (3.47%)	2 (2.86%)	10 (3.36%)
⩾10	227 (6.88%)	44 (4.49%)	28 (7.31%)	19 (4.68%)	21 (13.55%)	57 (3.48%)	39 (3.43%)	12 (2.63%)	4 (2.84%)	106 (8.26%)	76 (10.69%)	35 (8.29%)	2 (3.17%)	18 (14.75%)	3 (4.69%)	0 (0.00%)	33 (15.71%)	74 (11.92%)	20 (11.56%)	5 (7.14%)	38 (12.75%)
Total	3300	980	383	406	155	1636	1136	457	141	1283	711	422	63	122	64	89	210	621	173	70	298

LCS, low cervical segment; UTS, upper thoracic segment; MTS, middle thoracic segment; LTS, lower thoracic segment; CS, celiac segment.

Table S4. Cross-Table Analysis of Changes in the draft LNM diagnosis criterion to the redefined LNM diagnosis criterion of 393 nonsurgical patients with ESCC

Stage	r-negative	r-positive	Total
d-negative	5	0	5
d-positive	89	299	388
Total	94	299	393

d-x, the draft LNM diagnosis criterion; r-x, the redefined LNM diagnosis criterion

Table S5. Cross-Table Analysis of Changes in the draft nodal staging system to the novel LN clinical staging system of 393 nonsurgical patients with ESCC

Stage	n-N0	n-N1	n-N2	Total
d-N0	5	0	0	5
d-N1	63	58	53	174
d-N2	26	54	134	214
Total	94	112	187	393

d-Nx, the draft nodal staging system; n-Nx, the novel LN clinical staging system

Supplementary Figures

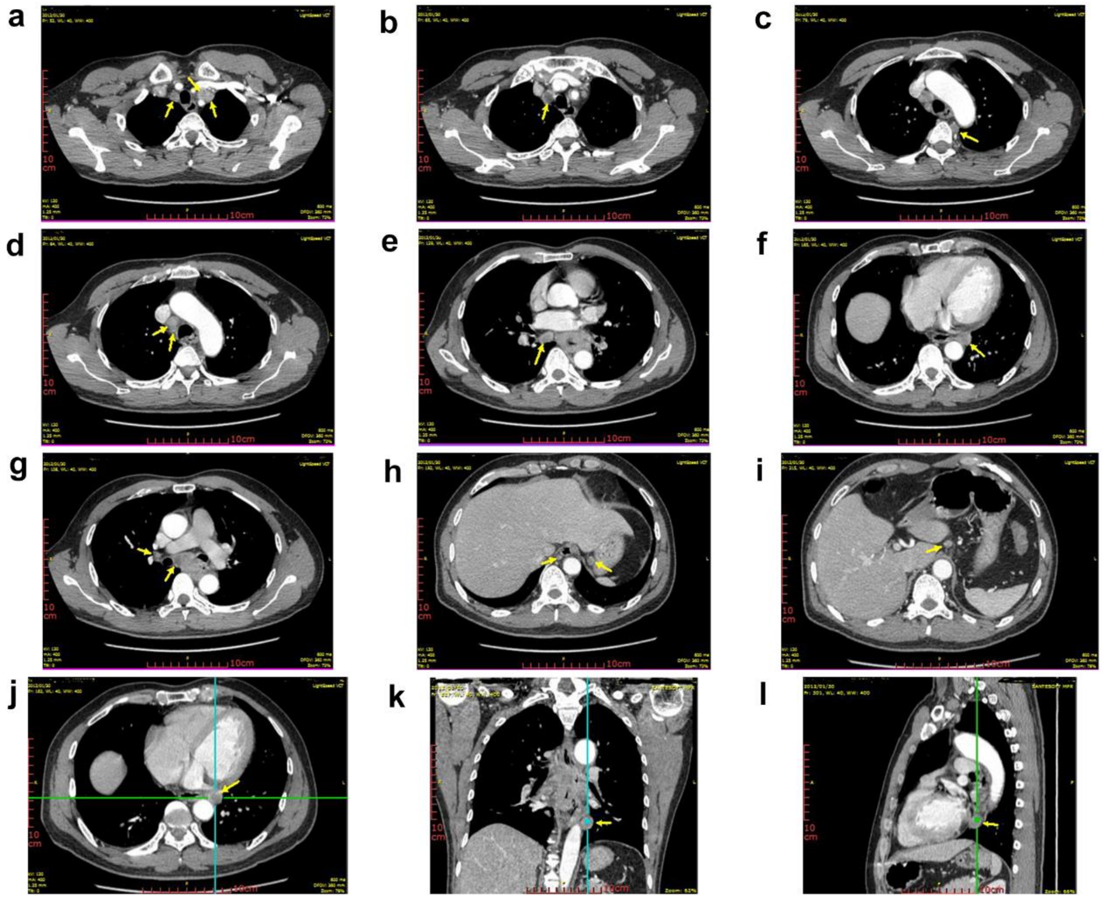

Figure S1. The MSCT images of a 45-year-old man with ESCC receiving nonsurgical palliative treatment.

(a) The size of 3 metastatic lymph nodes are 14.1x10.5 mm, 16.9x14.1 mm and 11.3x8.4 mm in the supraclavicular nodes station, respectively. (b) The size of the metastatic lymph node is 16.9x15.5 mm in the right upper paratracheal nodes station. (c) The size of the metastatic lymph node is 12.0x7.0 mm in the posterior mediastinal nodes station. (d) The size of 2 metastatic lymph nodes are 16.4x14.1 mm and 11.3x9.8 mm in the right lower paratracheal nodes station, respectively. (e) The size of the metastatic lymph node is 17.6x12.0 mm in the middle paraesophageal nodes station. (f) The size of the metastatic lymph node is 11.3x9.8 mm, 16.9x14.1 mm and 11.3x8.4 mm in the pulmonary ligament nodes station. (g) The size of 2 metastatic lymph nodes are 8.4x5.6 mm in the right tracheobronchial nodes station, and 20.4x12.7 mm in the subcarinal nodes station, respectively. (h) The size of 2 metastatic lymph nodes are 5.5x4.6 mm in the diaphragmatic nodes station, and 20.4x12.7 mm in the paracardial nodes station, respectively. (i) The size of the metastatic lymph node is 6.3x5.6 mm in the left gastric nodes station. (j, k and l) By axial surface, coronal section, sagittal place analysis, MSCT image can accurately display the metastatic lymph node in the pulmonary ligament nodes station.

The short and long-axis diameter of a lymph node in three dimensions were measured, as shown.

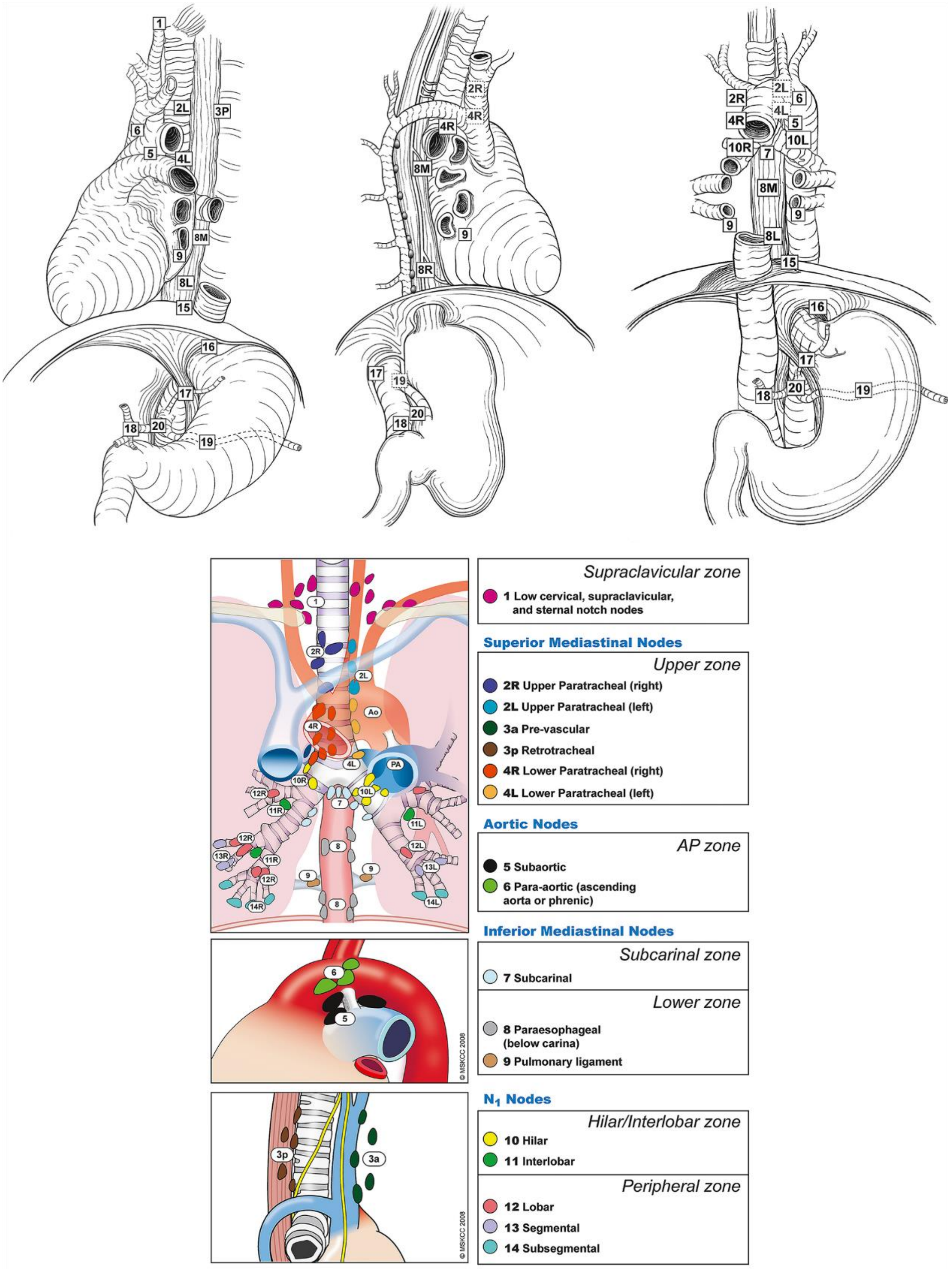

Figure S2. Lymph node sites defined by the IASLC lymph node map and Memorial Sloan-Kettering Cancer Center, 2009 for Esophageal cancer.

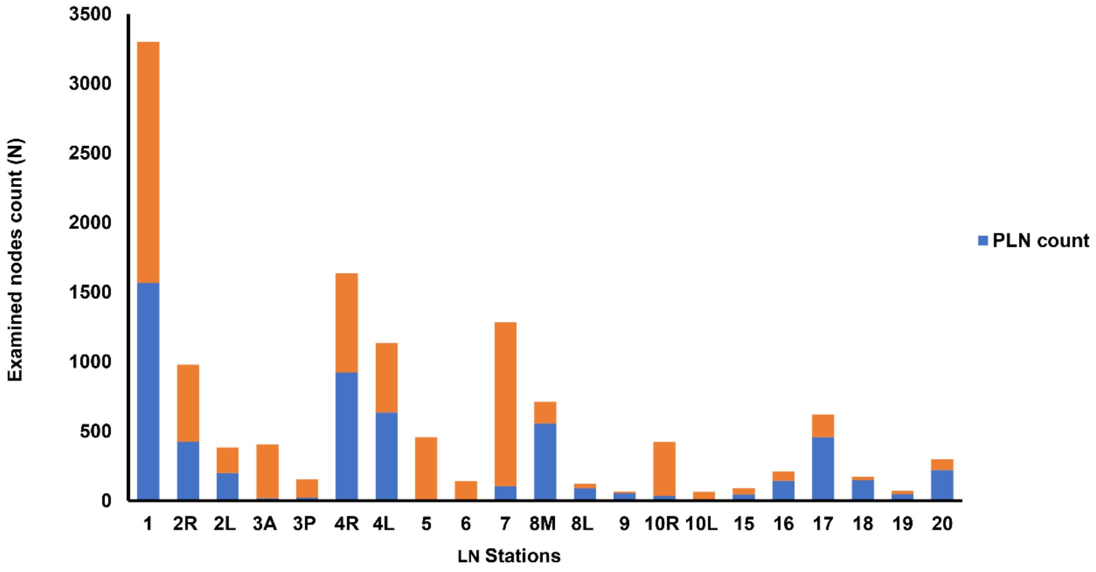

Figure S3. The distribution of patients with examined and positive lymph nodes in each station.

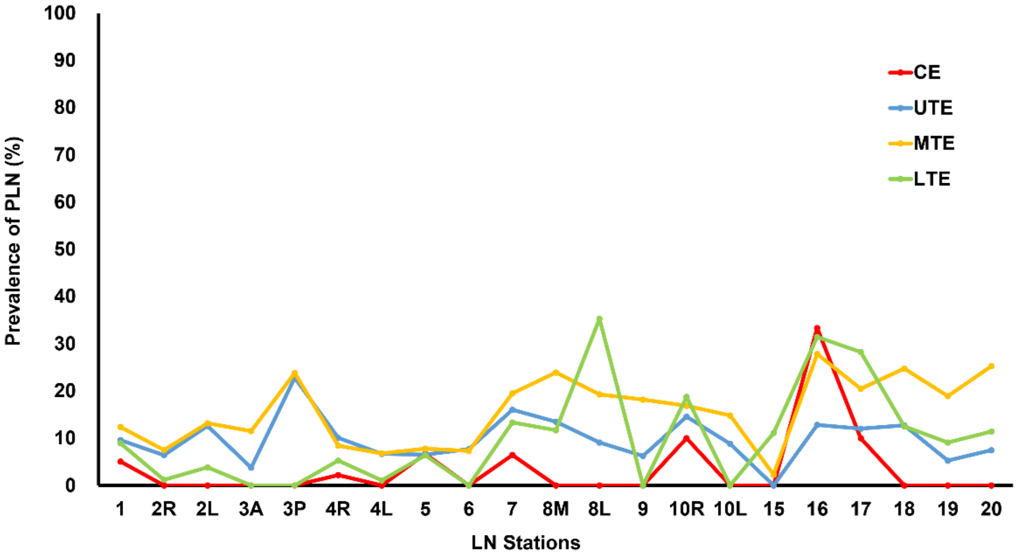

Figure S4. Multiple line chart of incidence of PLN in varying nodal stations separated by tumor location. The light gray, dark gray, black and red lines indicate tumor was located at CE, UTE, MTE and LTE, respectively. CE, cervical esophagus; UTE, upper thoracic esophagus; MTE, middle thoracic esophagus; LTE, lower thoracic esophagus.

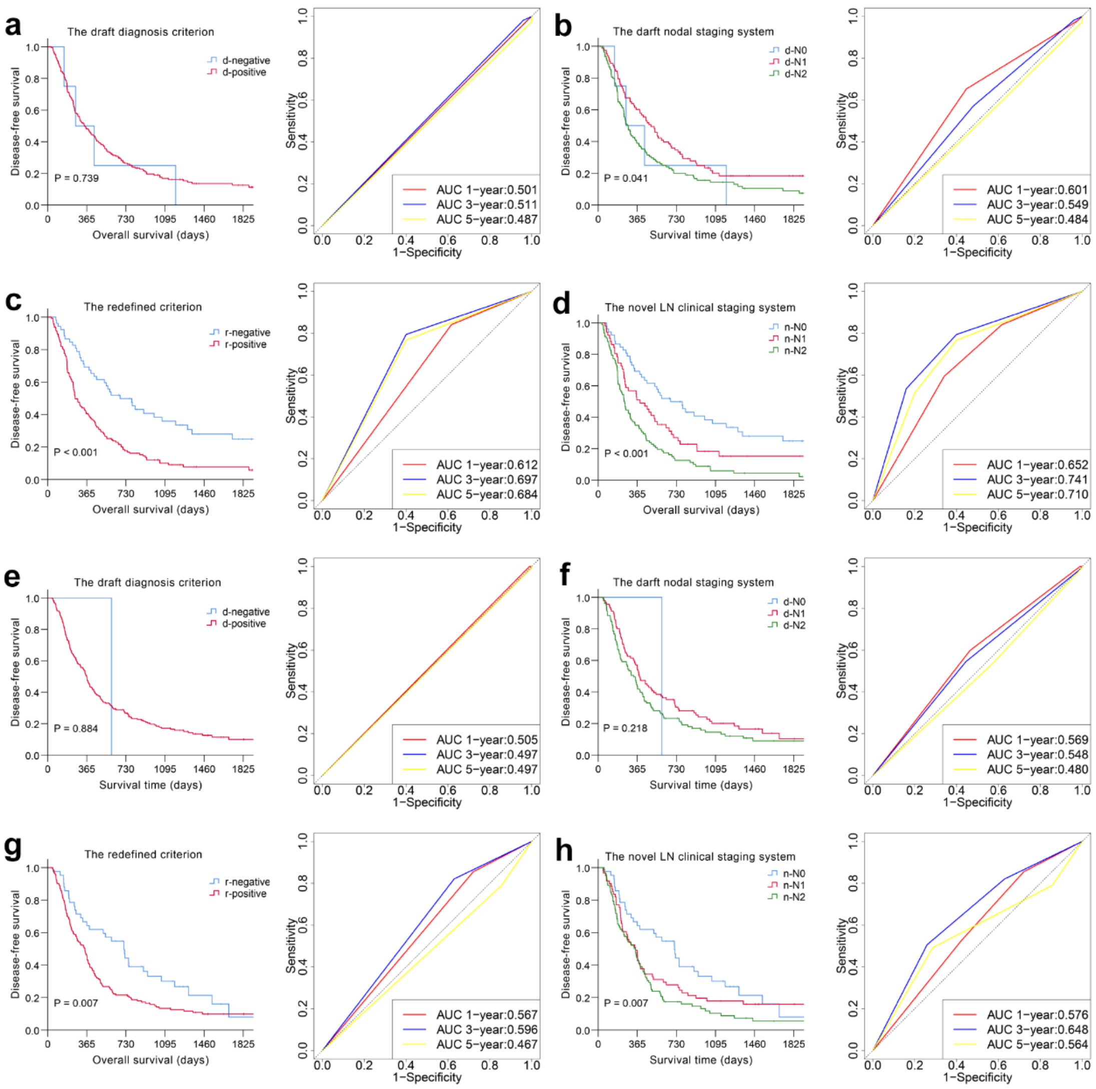

Figure S5. Disease-free predictive performance comparison of two diagnosis criteria for LNM and clinical staging systems were measured by the survival analysis and the time-dependent ROC curves in the derivation (a, b, c, d) and validation cohorts (e, f, g, h).

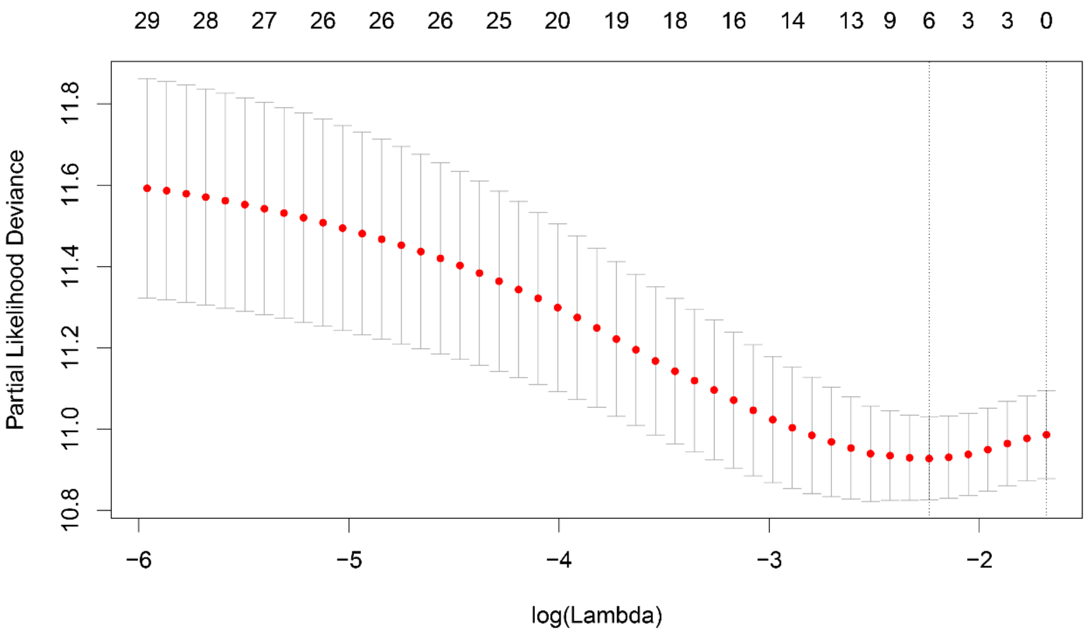

Figure S6. Five-time cross-validation for tuning parameter selection in the LASSO model.

Solid vertical lines represent partial likelihood deviance ± standard error (SE). The dotted vertical lines are drawn at the optimal values by minimum criterion and 1-SE criterion. We plotted the partial likelihood deviance versus log (λ), where λ is the tuning parameter. Herein, a value λ = 0.1066 with log (λ) = -2.239 was chosen by 5-time cross-validation via 1-SE criterion.

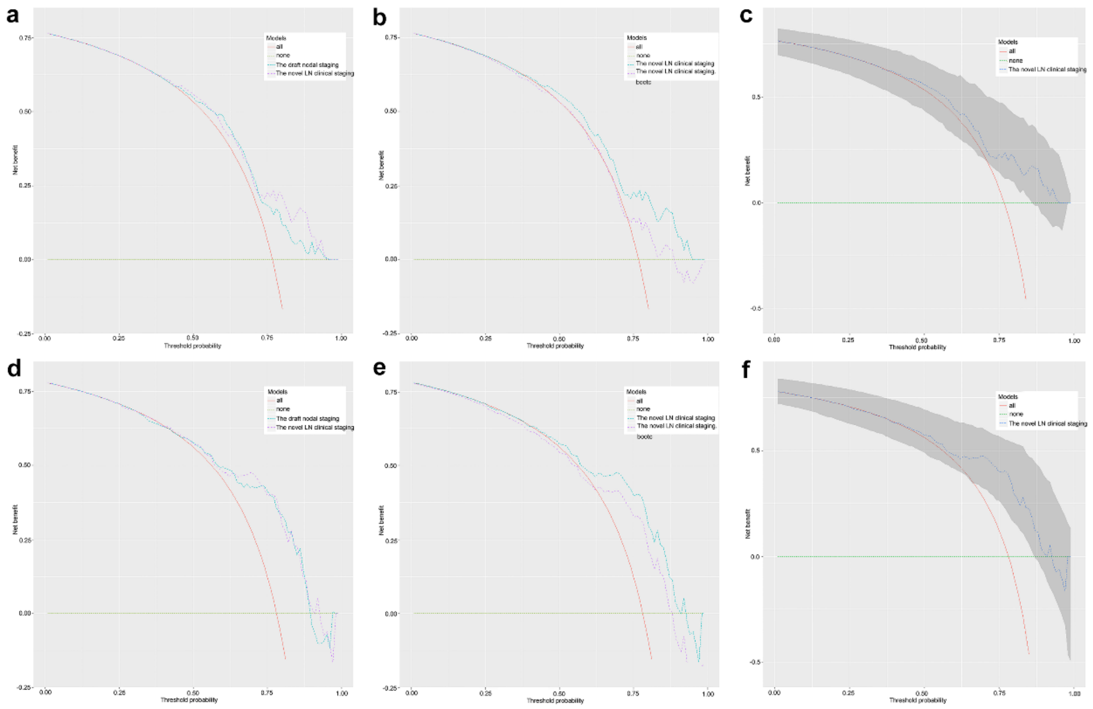

Figure S7. Decision curve analysis of the draft nodal staging system and the novel LN clinical staging system.

(a) Comparison of the practical clinical value by the draft nodal staging system and the novel LN clinical staging system in the derivation cohort. (b) Decision curve analysis with bootstrap corrected for the novel LN clinical staging system in the derivation cohort. (c) Decision curve with 95% confidence intervals for the novel LN clinical staging system in the derivation cohort. (d) Comparison of the practical clinical value by the draft nodal staging system and the novel LN clinical staging system in the validation cohort. (e) Decision curve analysis with bootstrap corrected for the novel LN clinical staging system in the validation cohort. (f) Decision curve with 95% confidence intervals for the novel LN clinical staging system in the validation cohort.

It turns out that the two clinical staging systems significantly differ at the 5% level for 11 threshold probabilities in the derivation cohort while they did not significantly differ at the remaining 88 thresholds. In the validation cohort, the two clinical staging systems significantly differ at the 5% level for 8 threshold probabilities while they did not significantly differ at the remaining 91 thresholds.

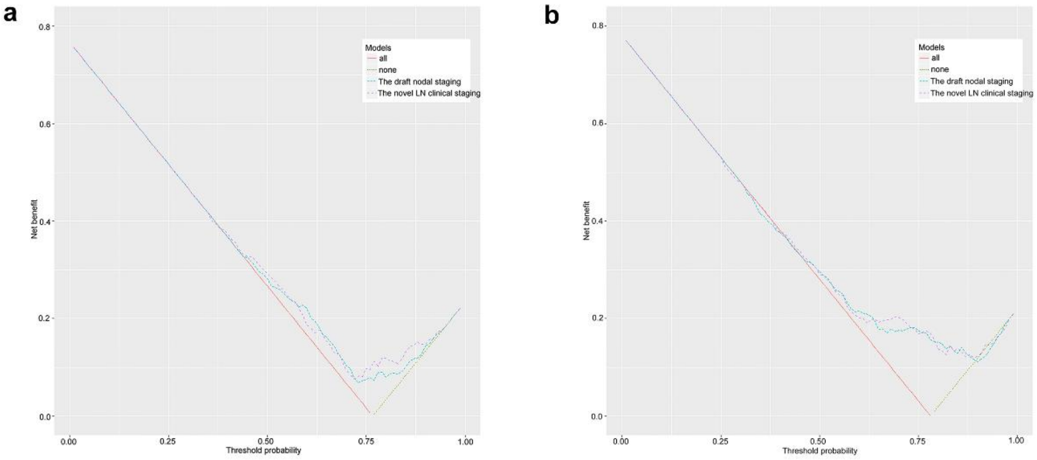

Figure S8. ADAPT curves for the draft nodal staging system and the novel LN clinical staging system. (a) Comparison of the ADAPT value by the draft nodal staging system and the novel LN clinical staging system in the derivation cohort. (b) Comparison of the ADAPT value by the draft nodal staging system and the novel LN clinical staging system in the validation cohort. ADAPT, average deviation about the probability threshold.
